# Implementing TeleSleep at Veterans Healthcare Administration: an organizational case study of adaptation and sustainment

**DOI:** 10.3389/frsle.2024.1444689

**Published:** 2024-09-11

**Authors:** Jeffrey K. Belkora, Jill Reichert, Katherine Williams, Mary A. Whooley, Talayeh Rezayat, Stacy Sorensen, Priyanka Chilakamarri, Elizabeth Sanders, Andrea Maas, Alexander Gomez, Philip Kurien, Liza Ashbrook, Jacque Thomas, Kathleen F. Sarmiento

**Affiliations:** ^1^Institute of Health Policy Studies and Department of Surgery, University of California, San Francisco, San Francisco, CA, United States; ^2^San Francisco Veterans Affairs Health Care System, San Francisco, CA, United States; ^3^Department of Medicine, University of California, San Francisco, San Francisco, CA, United States; ^4^Department of Medicine, University of Nevada, Reno, Reno, NV, United States; ^5^Department of Neurology, University of California, San Francisco, San Francisco, CA, United States; ^6^Department of Anesthesia, University of California, San Francisco, San Francisco, CA, United States

**Keywords:** sleep medicine, telehealth, implementation, adaptation, sustainment, program process theory, program planning, organizational case study

## Abstract

Veteran access to sleep medicine is of paramount importance to the Veterans Health Administration (VA). To increase access, VA has created community referral policies and programs, as well as telehealth programs. In 2017, the Office of Rural Health (ORH) funded a TeleSleep initiative focused on reaching rural Veterans with unmet sleep needs. ORH provided 3–6 years of funding to help 19 hubs support 98 spoke sites serving rural Veterans. As ORH funding concluded, each hub identified its path to sustainment. This case study follows one TeleSleep hub in VA's western geographic region as it transitioned from ORH funding sustainment as a regional Sleep Clinical Resource Hub. This case study describes the real-world process of adaptation in care delivery strategies. One key area of adaptation revolved around whether to deliver care via the patient's home facility or the provider's home facility. In early 2021, the TeleSleep team implemented an innovative provider transfer model, where temporary reinforcements from the TeleSleep hub increased the workforce capacity of spoke sites, similar to the concept of locum tenens. In this provider transfer model, TeleSleep clinicians scheduled, documented, and billed for each encounter at the Veteran's home facility. Positioning TeleSleep clinicians as local providers facilitated communication and referrals and promoted continuity and quality of care for Veterans in their home facility. This provider transfer model reduced the administrative burden of providers and schedulers and supported patient-side-only documentation of care. While this mirrors current locum tenens practice, transferring providers did not fit VA's financial model as implemented by the western region's Sleep Clinical Resource Hub. Therefore, in December 2021, VA aligned TeleSleep with VA's preferred practice of patient rather than provider transfers. In the patient transfer model, providers schedule and document in both the provider and patient electronic health records, and bill in the provider's facility. However, reflecting on this period of innovation, TeleSleep team members concluded that the provider transfer model could improve patient safety and care coordination while reducing the administrative burden of frontline clinicians. Further research and development are needed to align the provider transfer model with VA's financial model.

## 1 Introduction: description of the nature of the problem being addressed and rationale for the proposed innovation

Veterans suffer disturbed sleep due to conditions ranging from sleep apnea to post-traumatic stress disorder. In response to increasing Veteran demand for comprehensive sleep services, the Veterans Health Administration (VA) seeks to grow capacity by expanding facility-based services and telehealth infrastructure (Kaul et al., 2021; Sarmiento et al., 2019).

For telehealth delivery of sleep care, VA initially targeted rural regions, which are more likely to be designated as medically underserved. VA's Office of Rural Health (ORH) invested in a successful delivery model, the TeleSleep Enterprise-Wide Initiative, beginning in March 2017. This initiative provided initial funding for hub (usually urban medical centers) and spoke (rural or highly rural) sites to collaborate on virtual delivery of sleep care (Chun et al., 2023). In the ORH initiative, 19 rural-serving facilities with 98 spoke sites volunteered to be early adopters of TeleSleep, and the implementation was successful in reaching Veterans and increasing access to sleep care (Sarmiento et al., 2019).

In April 2020, the Sierra Pacific Veteran Integrated Service Network (VISN 21), consisting of seven VA healthcare systems with 50 spoke sites in the western region of the United States, concluded that virtual sleep medicine could benefit non-rural as well as rural Veterans. VISN 21 provided financial support for a San Francisco-based TeleSleep hub with the goal of providing services regionally. This implementation case study provides a detailed account of how TeleSleep adapted as it evolved from ORH funding to VISN 21 sustainment.

## 2 Context (setting and population) in which the innovation occurs

The setting for this case study was VISN 21, described by VA as follows: “The Network serves Veterans in northern and central California, Nevada, Hawaii, the Philippines and U.S. Territories in the Pacific Basin. VISN 21 provides a continuum of comprehensive health care services through seven VA Medical Centers, nine co-located Community Living Centers, and 41 Community-Based Outpatient Clinics (CBOCs).” (Veterans Health Administration, 2021).

VISN 21 serves over 400,000 Veterans, many of whom suffer from disordered sleep. In 2019, sleep medicine doctors in VISN 21 tested or treated 24,000 Veterans, mostly through in-person care pathways. The prevalence of diagnosed sleep-related breathing disorders among Veterans treated at the VA was 22% in 2018, with an actual estimated prevalence exceeding 60% when including undiagnosed Veterans (Folmer et al., 2020). Thus, we estimate the VISN's total addressable population for sleep medicine as in the range of 88,000 (22% of 400,000) to 240,000 Veterans (60% of 400,000).

## 3 Case study time frame

**Figure 1:**
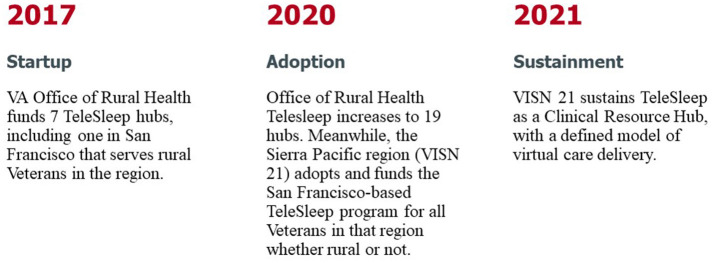
Evolution of TeleSleep from startup through adoption to sustainment.

This case study reports on the initial TeleSleep ORH program launched in March 2017 and follows it through the third year of sustainment as a VISN 21 Sleep Clinical Resource Hub in September 2023 (Figure 1).

## 4 Detail to understand key programmatic elements

### 4.1 Program planning framework

As we adapted TeleSleep, we relied on a five-step program planning framework (Collins, 1994; Rossi et al., 1999). This framework describes a program's internal processes (Belkora et al., 2023; Belkora, 2015). Following this framework, we continually refined TeleSleep's strategic; service; operational; financial; and evaluation plans (Table 1).

**Table 1:** Program processes.

	**TeleSleep processes**				
	**Strategic plan**	**Service plan**	**Operational plan**	**Financial plan**	**Evaluation plan**
Description	High-level (strategic) objectives	Customer or end-user journey	Organizational actions to support customer journey	How to sustainably align program costs and revenues	Measures, outcomes, and impact
Examples	i. Increase Veteran access to sleep care ii. Provide sleep care reinforcements to facilities that need extra capacity	i. Veteran schedules and attends sleep care appointment ii. Veteran absorbs, understands, and acts upon advice, referrals, and resources provided	i. Cultivate and nurture referrals to TeleSleep ii. Build service capacity iii. Deliver services to Veterans	i. Virtually transfer providers to Veteran home facilities for seamless continuity of care ii. Reimburse provider home facility for provider time delivering care outside home facility	i. Adjust attribution of fixed indirect costs to reflect that TeleSleep providers mostly work from home. ii. Adjust calculation of provider productivity to adjust for lack of clerks and medical assistants iii. Recognize how virtual care contributes to VA global objectives that are not always valued locally

### 4.2 Strategic plan

In 2020, upon being adopted by the VISN 21 region, the San Francisco-based TeleSleep hub drafted a strategic plan. We first articulated a problem statement: “Sleep disorders and poor sleep quality affect the quality of life of millions of Veterans and contribute to serious illness, disability and even death.” In the face of these problems, we stated the TeleSleep vision: “Save lives and reduce suffering by creating a world in which Veterans sleep all night, every night.” Toward this vision, we asserted the TeleSleep purpose: “Deliver sleep care to Veterans wherever and whenever they need it—in a way that is financially sustainable for VA.” TeleSleep mobilized around a mission statement: “Reduce disparities in access to sleep care among Veterans.” We summarized our initial approach: “Provide sleep care reinforcements to facilities that need extra capacity.” Our tagline: “Better Sleep, Better Health.” Measurable goals and objectives for TeleSleep included: increase VISN 21′s capacity to provide sleep care to its Veterans; stabilize the workforce within VISN 21; provide gap coverage for providers on extended leave or upon staff turnover; improve care coordination for Veterans; reduce avoidable referrals to outsourced community care; and reduce overall costs of community care (Kaul et al., 2021; Donovan et al., 2019; Weaver et al., 2020).

In June 2021, VA faced the global safety recall of positive airway pressure devices used by more than 700,000 Veterans. This required TeleSleep leaders to adapt and address an additional set of sleep care needs for impacted Veterans (Belkora et al., 2023). The recall spurred innovation in delivering TeleSleep care with the goal of getting patients scheduled and seen as quickly as possible to support treatment decisions involving affected devices. To accomplish this goal, VA opened recall clinics each facility and assigned TeleSleep clinicians to staff these clinics. These providers scheduled, documented, and billed at the patient's home facility. Another strategic adaptation occurred in December 2021, when VISN 21 completed the integration of the TeleSleep program into VISN 21′s Clinical Resource Hub (CRH) model of virtual care delivery (Rubenstein et al., 2023; Gujral et al., 2024; Burnett et al., 2023).

### 4.3 Service plan

We characterized the baseline (in-person) sleep medicine service plan in a Veteran-centered narrative, illustrated by quotes from earlier qualitative research (Nicosia et al., 2021) (Table 2). This is consistent with the VA's strategic initiative to describe services in terms of Veteran life journey maps (US Department of Veterans Affairs, 2022).

**Table 2:** Veteran journey through diagnosis and treatment for sleep disturbances—steps and description.

1. Veteran experiences sleep-related health issues	These issues may include sleep disordered breathing, insomnia, shift work disorder, and trauma-associated sleep disruption.
2. Veteran notices sleep-related health issues	“I was waking up three or four hours a night, my husband couldn't sleep…” (Nicosia et al., 2021)
3. Veteran attends primary care or other visit at a VA facility, in person or virtually.	Prior to telehealth, Veterans attended primary care visits in person. COVID-19 accelerated adoption of telehealth for such visits.
4. Veteran discusses sleep-related health issues with VA provider	In a whole health approach, providers should query patients about their sleep to proactively elicit barriers to quality sleep.
5. Veteran acts upon advice and referrals from VA provider	Challenges at this step include how providers can best communicate with Veterans about the relevance of care and the treatment plan.
6. Veteran obtains specialty sleep care appointment	Ideally, the Veteran self-schedules this appointment; otherwise a VA employee contacts them. If wait times are beyond 28 days, VA must offer the Veteran the option to obtain care in the community (Kuna, 2019).
7. Veteran attends specialty sleep care appointment	Veterans may experience barriers to attending sleep appointments. For in-person visits, VA may reimburse for travel, but distance and time are still barriers: “I can't take transit because of the oxygen I have to carry. I can't drive because of my sleep problem. So, my brother-in-law has to take time to take me down there and we have to spend the night and it is just a hardship.” (Nicosia et al., 2021) In cases where sleep care is not available at the Veteran's VA facility, VA may refer the Veteran to care delivered by non-VA providers in the Veteran's community. “We had huge billing issues because they billed it incorrectly and they came out and said we owed $8,000 and I had to fight that in order for it to be billed correctly so the VA would pay for it.” (Nicosia et al., 2021)
8. Veteran acts upon advice and referrals from sleep care specialists	As for Step 5, providers and Veterans face the challenge of communicating about complex testing and treatment choices.
9. Veteran implements sleep care plan	As Veterans implement the care plan, they may encounter additional hurdles to accessing evaluation and treatment. As one Veteran stated, “The VA put me through CHOICE [outsourced care in the community]. I never got a machine. Because it just went around and around and around.” (Nicosia et al., 2021)
10. Veteran monitors and reports on sleep care progress	Having implemented a sleep care plan, ideally Veterans will monitor and report on their progress.
11. Veteran attends follow-up visits and engages in course-correction	Once care is established, Veterans should continue to engage with their care providers so that they can adapt the care plan based on progress or problems. This requires open communication and follow-through from both providers and Veterans.
12. Veteran experiences sleep-related health improvements	After implementing prescribed sleep care strategies, Veterans can experience day to day health and quality of life benefits. “Since starting sleep care, I am out like a light. Instantly. It's nice. It's been great.” … “But now with the CPAP machine, no foolin', for the last year we have been in bed just about every night together, snuggling, and I mean it's like, this is wonderful.” (Nicosia et al., 2021)
13. Veteran maintains sleep care	As with any intervention, the Veteran must maintain their efforts to sustain the benefits. At a minimum, VA must maintain the Veteran's devices and connections or otherwise support the Veteran with ongoing sleep interventions.

The initial ORH TeleSleep program aimed to improve the journey for rural Veterans by tapping into the telehealth capacity of sleep providers at various VA facilities. Specifically, TeleSleep introduced an optional virtual referral to specialist sleep care at steps 5 and 7 in the Veteran's journey (Table 2). In step 7, sleep technologists, respiratory therapists, or telehealth clinical technologists taught Veterans to use specialized sleep recorders at their domicile. TeleSleep physicians and nurse practitioners then interpreted the test results and prescribed therapies as needed.

For steps 8–10 of the Veteran journey, TeleSleep referred Veterans to various services such as surgery, dental, behavioral sleep medicine, and weight management programs, or prescriptions for prosthetic devices or medications. Where possible, TeleSleep delivered care via telehealth.

From the Veteran's perspective, the TeleSleep journey remained similar for the implementations in 2017 by Office of Rural Health and in 2020 by the Sierra Pacific Region (VISN 21). Veterans could always decline TeleSleep in favor of in-person care. For some Veterans, the journey became more complicated in June 2021 with the global safety recall of positive airway pressure devices. For those Veterans, we described changes to the Veteran journey in a published case study (Belkora et al., 2023).

### 4.4 Operational plan

The San Francisco-based TeleSleep hub articulated the following operational plan for its VISN 21 implementation in 2020.

#### 4.4.1 Scan the environment and define the plan

TeleSleep leaders identified stakeholders; took an inventory of readily available sleep resources at every facility within VISN 21; evaluated cost and volume of services referred to “community care” outside of VA; and defined project roles, responsibilities, and an organizational chart. Weekly meetings with facility sleep leads provided insight into the needs of each facility at the local level and established communications to begin standardization and dissemination of information.

#### 4.4.2 Coordinate personnel

TeleSleep coordinated its personnel through synchronous (e.g., chat, online meetings) and asynchronous communication (e.g., email). Through these mechanisms, TeleSleep onboarded and trained personnel in its procedures. Occasional in-person meetings also helped strengthen relationships and collaboration among team members.

TeleSleep also designed and implemented a provider coverage schedule across multiple clinic time zones (Pacific, Hawaii, Mountain, Samoa, Chamorro Time Zones), and allowed employees to live throughout these time zones as well as in Central and Eastern time. This geographic expansion increased the pool of qualified providers and facilitated recruitment. Being virtual, providers could also more easily cover for each other during absences.

#### 4.4.3 Cultivate and nurture referrals to TeleSleep

TeleSleep signed formal referral agreements with VISN 21 facilities. These agreements included Service Level Agreements and Telehealth Service Agreements, which cover credentialing and privileging, scopes of services, and guidance on referral and discharge criteria and how to request coverage by the VISN 21 TeleSleep team. TeleSleep leadership presented an overview of services to multiple stakeholder audiences including facility-based sleep staff, VISN 21 community of practice, and Community Care staff (who could offer virtual care by VISN 21′s TeleSleep team as an alternative to community care). TeleSleep programmed a VA informatics application (known as Light Electronic Access Framework) as a portal to solicit, receive, route, and track requests for telehealth services.

#### 4.4.4 Build service capacity

In order to provide comprehensive virtual sleep care across VISN 21, TeleSleep established several realms including behavioral, dental, medical, and surgical sleep subspecialty services. Within these services, TeleSleep hired clinical and administrative personnel. The clinical personnel included physicians, nurses, respiratory therapists, medical instrument technicians, dentist, and dental assistant. Administrative personnel initially included a program manager, business manager, and advanced medical support assistants. VISN 21 subsequently reassigned these administrative personnel outside of TeleSleep in 2021. Hiring and onboarding took longer than expected due to the following issues. First, both TeleSleep leadership and VISN 21 human resources personnel faced capacity constraints. Second, for VA's central human resources function, bringing new personnel into a VISN based structure was novel, and VA had to develop and gain experience with new procedures for doing so. Third, the VISN 21 Clinical Resource Hub could provide only very limited administrative support to TeleSleep during 2020–21.

Despite its virtual nature, the program still had to arrange dedicated space to house and teach health professions trainees, and to store, distribute and process sleep equipment. Trainees included sleep medicine fellows and internal medicine and neurology residents, who collectively further increased TeleSleep capacity. Clinical equipment included home sleep apnea testing (HSAT) and positive airway pressure (PAP) devices used in testing and treating sleep disordered breathing.

Delivering care began with building of clinic schedules for surgical, medical, and sleep testing appointments. Each facility in VA has its own instance of the electronic health record system; there are 170 instances in total and 8 instances in VISN 21. Prior to TeleSleep, these instances had no standardized interfaces (e.g., menus) or care pathways for sleep medicine. TeleSleep also had to configure systems for scheduling patients in remote facilities; for conducting telephone or video visits; for viewing and editing patient medical records; for viewing raw data or interpreting results from diagnostic or therapeutic interventions; and for prescribing treatments and ordering prosthetic or other devices.

#### 4.4.5 Deliver services to Veterans

In order to identify referrals, a TeleSleep consult manager queried a centralized consult tracker fed by each facility's health record system for electronic consults placed by referring providers. In following up on each referral, TeleSleep providers conducted comprehensive reviews of VA, Department of Defense, and non-VA community records in order to triage patients to the next step of care with appropriate context. They communicated the results of this triage to TeleSleep schedulers, with instructions regarding which scheduling grid was appropriate. These grids reflect the practitioner type (e.g., physician, nurse practitioner, respiratory therapist, etc.), availability of open appointments, modality of how the visit would occur (e.g., video chat vs. telephone), and reminder communication method (e.g., Short Messaging Service text, postcard, letter).

Schedulers booked TeleSleep appointments following two different scenarios, corresponding to the two major epochs in the program's evolution. Between March 2017 and December 2021, TeleSleep pioneered a new service delivery model. Before TeleSleep, when patients sought care remotely in person, the patient traveled to the remote facility, registered as new patients at that facility, and experienced care as if that facility were their home facility. When developing TeleSleep as an ORH program, program leaders introduced a patient-centered variation. For some participating sites, the ORH instead arranged to bring the remote providers into the Veteran's home facility as if that facility were the provider's home facility. For those sites, between March 2017 and December 2021, schedulers entered one appointment in the health record system of the Veteran's home facility. After 2021, when TeleSleep was fully integrated into VA's Clinical Resource Hub model, TeleSleep reverted to the Clinical Resource Hub practice of transferring Veterans to the provider home facility. Therefore, after 2021, schedulers needed to enter two mirrored appointments: one in the Veteran's home facility and one in the provider's home facility.

Once scheduled, TeleSleep providers delivered services to Veterans online, by telephone, by video chat, and by mail. For example, TeleSleep mailed sleep testing devices to Veterans; educated the Veterans by phone or video chat about sleep apnea and trained them on how to complete the test at their domicile; downloaded, scored, and processed returned devices; interpreted data to establish a diagnosis; notified patients of test results and the care plan; entered orders or consults for follow up care consistent with the care plan established or service agreement with the spoke facility. If the Veteran required a Positive Airway Pressure device, the TeleSleep provider prescribed it; ordered the device and supplies from a centralized warehouse; and educated the Veteran on use of the device and remotely monitored treatment to follow.

When documenting encounters and coding them for billing purposes, TeleSleep providers followed two different scenarios, corresponding once again to two different epochs in the program's evolution. Between March 2017 and December 2021, the provider created a single progress note in the electronic health record system of the Veteran's home facility. In addition to documenting the encounter, the remote sleep provider could add a return to clinic order. This alerted local schedulers to schedule a follow up appointment with the referring provider in order to assure continuity of care. The remote sleep provider also used the electronic health record at the Veteran's home facility to refer the Veteran to any required local services such as sleep testing. The remote provider documented all this in the electronic health record of the Veteran's home facility in order to maximize local visibility into the entire remote episode of care. Prior to December 2021, this Veteran-side documentation also included billing information for the episode of care.

After 2021, providers entered a progress note in the provider's home facility system that included billing information. To maintain full visibility in the Veteran's home facility system, the provider additionally documented in the Veteran's home facility record a full note with detailed clinical information as well as referrals and return to clinic orders. This Veteran-side documentation did not include billing information.

#### 4.4.6 Return veterans to facilities

When a Veteran completed an episode of testing and treatment, TeleSleep providers returned the Veteran to the referring providers in the Veteran's home facility. TeleSleep providers accomplished this by discussing a follow up care plan with the Veteran; writing a transition-of-care note that they asked a provider from the Veteran's home facility to additionally sign; and placing a “return to clinic” request for local facility schedulers to schedule the Veteran with the referring provider for follow-up care. That follow-up care often leveraged VA employees at the Veteran's home facility who were able to implement the care prescribed by the TeleSleep specialty care providers. Such employees extending the sleep care included sleep technologists, respiratory therapists, and registered nurses. These facility-based employees were able to support patients through sleep testing, treatment (e.g., Positive Airway Pressure devices), care coordination, and education.

### 4.5 Financial plan

While innovating in telehealth, TeleSleep had to navigate financial structures within VA designed for in-person, facility-based care. One constraint was that facilities pay salaries from budget allocations generated by provider workload, and VA providers can only be paid by a single facility, i.e., the provider's home facility. Meanwhile, VA assigns each Veteran to a home facility where Veterans ideally obtain as much of their care as possible to assure continuity and quality of care.

In conventional VA care delivery programs, when providers at a Veteran's home facility cannot deliver the care required by a Veteran, they can transfer the patient to a facility where care is available. This is known as an interfacility patient transfer. Veterans can also seek care in community settings, but for this report we are focusing on how VA can best provide care inside the system.

One challenge with interfacility patient transfers is that they result in episodes of care away from the Veteran's home facility. Veterans register as new patients in the remote facility, where providers document, bill, and refer Veterans onward in the same way they treat Veterans for whom this is the home facility. Once the remote episode of care is complete, Veterans usually resume care for other conditions in their original home facility. Providers in the Veteran home facility can in theory look up the remote episodes of care by either (1) navigating to the consults section of the medical record, locating the interfacility consult, and expanding it to reveal associated documentation; or (2) launching another online portal to review medical records and using search terms for domains of care of interest (e.g., “sleep”, “echocardiography”, “pulmonary function test”). In practice only primary care providers, who order most interfacility care, consistently look up remote episodes, which under current conditions is very time consuming. Often the specialty care providers in the Veteran's home facility resume care without awareness of the care provided in remote facilities. This can result in problems with continuity, safety, and quality of care.

From March 2017 to December 2021, to address these challenges, TeleSleep implemented interfacility provider (rather than patient) transfers. TeleSleep providers joined the Veteran's home facility as if they were locum tenens, or visiting providers, and documented care inside the Veteran's usual and ongoing electronic health record. During the period March 2017 to April 2020, the ORH reimbursed the provider facilities for work done at Veteran home facilities. For TeleSleep's implementation in VISN 21, starting in April 2020, TeleSleep providers were not eligible for this funding from the ORH. Instead, TeleSleep proposed that VISN 21 facilities receiving TeleSleep care transfer funds to purchase this care from the provider's facility. However, VISN 21 never implemented the funds transfer model. Rather, VISN 21 required TeleSleep to align itself with the Clinical Resource Hub model. This model uses interfacility patient transfers and all billing is done at the provider facility. In order to address the quality, documentation, and coordination of care concerns resulting from interfacility patient transfers, the Clinical Resource Hub implemented double documentation. Providers billed and documented episodes of care in the electronic health record system of the provider's home facility; and then re-documented in the electronic health record system of the patient's home facility.

### 4.6 Evaluation plan

VA generally evaluates its care delivery using measures of volume of care delivered (outputs); labor and other costs of care delivered (inputs); and quality (outcomes). When accounting for costs, VA attributes fixed and variable costs to production units such as sleep programs using methodologies developed for physical facilities.

However, virtual programs such as TeleSleep may incur very different fixed and variable costs than facility-based sleep programs. VA has not yet adjusted its attribution of fixed and variable costs to reflect the differences. For example, VA currently attributes high fixed indirect costs to virtual programs such as TeleSleep, even though TeleSleep employees actually bear many of these costs themselves (e.g., home office, internet, and electricity).

Likewise, when measuring volume of care delivered, VA uses methodologies developed for delivery of in-person care at provider home facilities (which may not be the Veteran's home facility, as described above). In physical facilities, VA focuses on the length and number of visits as measures of production for providers. It's worth noting that, in physical facilities, staff are available to streamline patient visits, which increases provider productivity. For example, in physical facilities, clerks and medical assistants take vital signs, room patients, administer questionnaires, and complete mandatory health screenings. Furthermore, in person visits, rooming time is not included in the calculation of how long the visit lasts. Providers see a queue of Veterans in the order that they are roomed, and the visit time starts when the provider enters an exam room where a Veteran is waiting.

Conversely, virtual providers often conduct online visits without staff assistance. Providers often wait while Veterans navigate unfamiliar technology to enter the online appointment. This extra time corresponds to the in-person “rooming time” normally undertaken by medical assistants. In contrast to in person visits, here the Veteran's “rooming time” prolongs the appointment and VA evaluates this as reduced provider productivity. Providers also have to use visit time to administer questionnaires and screens and perform other tasks that are conducted by staff when visits are in person. Then providers still have to take history and guide patients in medical decision making. As a result, due to tasks being shifted from staff to providers, virtual providers take longer and see fewer patients.

VA's current view of virtual visits therefore is that they have similar costs to in-person visits (due to VA attributing similar fixed costs); while virtual providers complete a lower volume (due to providers waiting for Veterans to room themselves and providers using visit time to perform staff functions such as administering questionnaires and screens). Therefore, VA often perceives virtual care as costing more per unit of care delivered.

## 5 Discussion

As we trace the evolution of TeleSleep through this case study, we see variation in our five program dimensions: strategic plan, service plan, operational plan, financial plan, and evaluation plan. We can locate the variation on a spectrum ranging from VA's standard model of in-person, facility-based care to what we believe will be VA's future model of virtual provider transfers. We summarize the variation we observed in Table 3 and elaborate below.

**Table 3:** Redesigning the delivery of sleep medicine in the Veterans Health Administration.

**Plan**	**1. In-person care with option for interfacility patient transfer**	**2. Interfacility patient transfer for virtual care from facility-based provider**	**3. Patient transfer to virtual clinical resource hub**	**4. Virtual provider transfer to veteran's home facility**
Strategic	In-person care at a VA facility, with outsourcing to community when necessary	Adds option to in-source to virtual care elsewhere in VA if Veteran agrees		
	Each facility plans its own capacity		Requires cross-facility capacity planning	
	Facilities vary in how they implement sleep medicine programs (e.g., different processes used to refer patients for sleep services)		Standardization of sleep medicine processes across facilities (e.g., sleep study always ordered by a sleep specialist)	
Service	Veteran receives care in home facility or travels to other VA or community care site	Veteran uses VA Video Connect and other VA telehealth platforms to receive care virtually		
Operational	Requires adequate staffing for in-person visits at each facility	Allows for unplanned overflow from one VA facility to another	Virtual care plans for variation in demand from referring facilities with distributed dynamic staffing	
	Remote providers document in their home systems only		Remote providers document in both provider and Veteran home system	Remote providers document in Veteran home system only
	Potential loss of safety and quality when remote episodes of care are not easily visible in Veteran's home system		Safety gained but efficiency lost under dual documentation	Safety and efficiency gained when documenting in Veteran system
Financial	Provider's home system, not Veteran's home system, accrues budget based on this episode of care.			Veteran home facility accrues budgetary credit from virtual episodes of care
	Provider's home facility funds provider time for care provided to the Veteran.			Veteran's home facility funds provider time for virtual care provided to the Veteran
Evaluation	VA attributes fixed and variable costs to production units such as sleep programs in physical facilities. These attributions, combined with volume, drive the evaluation of efficiency (costs per unit of care).		VA attributes high fixed costs to virtual care but virtual employees bear many of these costs themselves (e.g., internet, electricity). Virtual hubs may appear to be as costly as physical facilities, even as they may be reducing overall cost and adding value to VA as a system.	

### 5.1 Strategic plan

**Figure 2:**
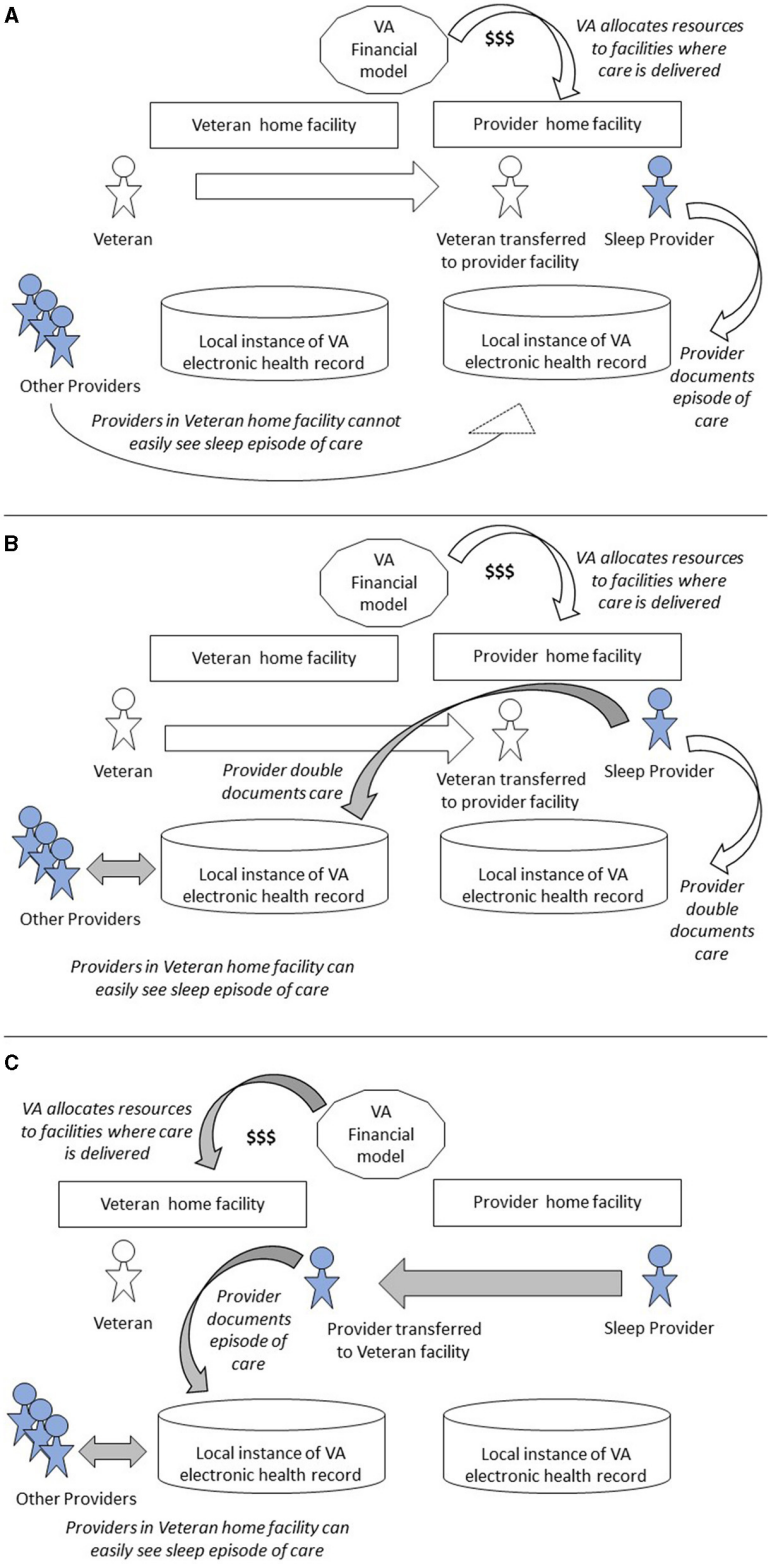
Variation in program processes when transferring patients vs. providers. **(A)** Patient transfer for in-person or Telehealth care. **(B)** Patient transfer to virtual Clinical Resource Hub. **(C)** Provider transfer to Veteran's home facility. Shaded arrows denote process changes, shaded people denote providers.

Each column of Table 3 represents a different strategy for assuring VA can provide care to all Veterans. Figure 2 presents a graphical illustration of key elements in Table 3.

#### 5.1.1 In-person care with option for interfacility patient transfer

Column 1 of Table 3 represents in-person care by sleep medicine providers. This is the traditional, legacy model of care in VA. If a Veteran's home facility lacks capacity to deliver timely access to care for the Veteran, the facility can transfer the patient to another facility for in-person care there. Figure 2A depicts this patient transfer scenario. VA pays to reimburse the Veteran for travel costs.

#### 5.1.2 Interfacility patient transfer for virtual care from facility-based provider

Column 2 represents the situation where facility-based providers occasionally deliver care via telehealth rather than in person, in order to help another facility meet overflowing Veteran needs. Figure 2A also captures this scenario, except that the care is delivered via telehealth rather than in person.

#### 5.1.3 Patient transfer to virtual Clinical Resource Hub

Column 3 represents a recent VA innovation, where a region creates a virtual Clinical Resource Hub. A Veteran's home facility can transfer the Veteran to this Clinical Resource Hub for care from a virtual team organized to deliver comprehensive sleep medicine as a regionally shared service. Figure 2B depicts this patient transfer scenario, in which providers document in the electronic health record systems of both provider and Veteran home facilities.

#### 5.1.4 Virtual provider transfer to Veteran's home facility

Column 4 represents a proposed future state where VA deploys locum tenens providers as a shared service and transfers providers, not patients, between facilities. The locum tenens providers attend to Veterans in the Veteran home facility via telehealth. Figure 2C depicts this provider transfer scenario.

### 5.2 Service plan

While each column of Table 3 represents a distinct strategy, the recent virtual care strategies all share a common service plan.

#### 5.2.1 Column 1, in person care with option for interfacility patient transfer

In this model, if the Veteran's home facility cannot provide timely access to sleep care for the Veteran, the Veteran must travel to a community site, or to another VA facility to obtain care. This is the legacy model that pre-dates telehealth.

#### 5.2.2 Columns 2–4, representing variations in care delivered virtually

In all variations of virtual care at VA, Veterans can make use of VA's robust telehealth infrastructure. This includes the possibility of using telehealth technology from the Veteran's home facility if the Veteran needs assistance, or logging in from their domicile if the Veteran has internet access and the requisite technology skills.

### 5.3 Operational plan

Each care strategy in Table 3 has a corresponding operational plan.

#### 5.3.1 Column 1, in person care with option for interfacility patient transfer

From an operational point of view, VA's legacy model faces challenges related to capacity planning. More Veterans require sleep care each year, but planners cannot predict exactly how many Veterans will seek such care from a facility in any given year. Planners must also contend with shortages in the supply of specialized labor; and competition to recruit and retain sleep medicine providers. The uncertainty and fluctuations in demand, combined with overall labor shortages, mean that facilities cannot always meet the regulatory standard for providing their Veterans with timely access to sleep care. The legacy VA model is to send the patient traveling in person to a community care site, or to another VA site with the capability to provide timely care. Transferring the patient to another facility allows VA as a system to in-source or out-source the provision of overflow care on an *ad-hoc*, unplanned basis.

#### 5.3.2 Column 2, interfacility patient transfer for virtual care from facility-based provider

With the advent of telehealth at VA, if a provider at the other facility is able to provide virtual care, they will do so. Virtual care avoids the costs and inconvenience of Veteran travel. However, whether providing care in person (above) or virtually, the remote provider documents in the provider's home system. This means that these remote episodes of care are not easily visible to providers in the electronic health record of the Veteran's home facility. This results in a potential loss of safety, quality, and continuity of care.

#### 5.3.3 Column 3, patient transfer to virtual Clinical Resource Hub

A virtual Clinical Resource Hub represents VA's planned/anticipated response to in-sourcing of overflow care. Ideally, the Clinical Resource Hub assembles a multidisciplinary team of providers who can deliver care wherever it is needed in the region. Providers in Clinical Resource Hubs deliver and document care in their home facilities, not the Veteran's home facility. However, they also document in the Veteran's home facility so that their episodes of care are easily visible to other providers in that facility. This double documentation is costly and wasteful in terms of provider time and morale, which also affect VA's challenges with retention and recruitment.

#### 5.3.4 Column 4, virtual provider transfer to Veteran's home facility

Transferring providers to the Veteran's home facility could be implemented as part of a VA movement toward a nationally distributed workforce. One advantage of this model is that providers deliver and document care only in the Veteran's home facility, and this episode of care is easily visible to other providers in the Veteran's home facility.

### 5.4 Financial plan

The first three strategies in Table 3 share a common financial plan, while the last requires financial innovation at VA.

#### 5.4.1 Columns 1–3, representing patient transfer models

When VA transfers Veterans internally, whether for in person or telehealth care, the provider accounts for the episode of care inside the provider's home facility, which is where VA deems the Veteran to be receiving care because the Veteran registers at that site to be seen by that provider. By the rules of VA's internal resource allocation system, known as the Veterans Equitable Resource Allocation or VERA, VA funnels resources to VISNs. VISNs in turn apply an allocation model to distribute funds to facilities in proportion to the population they serve and the care they have provided recently. Since VA rules also state that providers can only be paid by one facility, this system ensures that resources flow to where they are needed to pay providers. Note that VA designed and implemented VERA in an era of in-person care, i.e., before the advent of telehealth.

A consequence of this model, which is predicated on a capitated payment system, is that facilities that cannot deliver sleep medicine will cede budget to other facilities when referring Veterans to those facilities. This further erodes the capacity of the referring facilities. Yet VA also wants to maintain the capacity of facilities closest to the Veteran so that Veterans can obtain care near their domiciles. The dynamics of the current system will inexorably lead to concentration of resources at the largest urban facilities.

#### 5.4.2 Column 4, virtual provider transfer to Veteran's home facility

In our proposed future-state model, transferring the provider rather than the patient would allow the provider to deliver, document, and bill for care in the Veteran's home facility. Under existing VA accounting rules, the Veteran's home facility would get credit for such episodes of care. One implementation of this model could involve modifying VA rules so that providers could be paid by multiple facilities. All the facilities where a provider delivered care would contribute a pro-rata share of the provider wages. Alternatively, VA could allow the home facility to invoice other facilities for services delivered virtually by their providers. Regardless of the tactical details, the desired outcome would be to concentrate resources according to the location of the Veteran receiving care rather than the location of the provider delivering care.

### 5.5 Evaluation plan

We propose that VA should adapt its evaluation of care efficiency and effectiveness, as its current methods distort the actual costs of virtual care.

VA financial models arose before the advent of telehealth and allocate costs as if all care is delivered in person at physical facilities. As a result, VA still attributes high indirect costs to sleep care delivered virtually, even though purely virtual providers working at home pay for their own office space, internet and electricity. In addition, virtual care may be associated with cost savings to VA as a system, for example because of avoided community care costs or avoided travel costs for Veterans. By adjusting its calculations, VA might discover that virtual care incurs a lower unit cost than in-person care.

As for quality of care, VA may need to more explicitly value and recognize how virtual care contributes to VA strategic objectives including: increasing care coordination and quality for Veterans who stay in VA; reaching rural and highly rural Veterans who may be living in care deserts; retaining Veterans and providers in VA longitudinally; and avoiding costs of community care. Taking into account these considerations, virtual programs may be reducing overall cost and adding currently underrecognized value to VA as a system.

### 5.6 Limitations

As an implementation case study, our account relies on details relating to a specific context: a region in the Veterans Health Administration. The insights from our case study may not generalize to other health delivery systems. While we provided many implementation details related to telehealth delivery of sleep medicine, the scope of our report did not include how remote providers prescribe medications, including controlled substances often used in sleep medicine. Another issue that we deemed out of scope for this report was VA's implementation of a new electronic health record system. This system, when eventually implemented, may alleviate some of the concerns we raised about the visibility of remote episodes of care to providers in the Veteran's home facility. Meanwhile, the scope of our report included a detailed account of our proposal to transfer providers rather than patients. We recognize that this model has not been fully tested, and should ideally be piloted before broader implementation. One empirical question is how funding hub provider time at remote or spoke patient facilities would affect activities outside of patient care, such as training and research. Funds accruing to spoke facilities should increase their capacity for delivering care under VA's financial model, but in so doing would divert funds from the hub facilities where providers are based. A question arises as to whether this would erode the development of centers of excellence in hub facilities.

## 6 Conclusion

This organizational case study follows TeleSleep through multiple adaptations as a VA region adopted, implemented, and sustained the program. The latest implementation of TeleSleep has increased Veteran access to sleep care. In order to further improve quality of care, we recommend that VA transfer providers, not patients, between facilities so that providers can deliver virtual care as if they were in the patient's home facility. Transferring providers may require VA to adapt its financial management practices so that a provider's home facility gets reimbursed for any provider time spent delivering care to patients at another facility. VA could even consider creating a single administrative entity acting as a national hub for all TeleSleep services. Such a national TeleSleep hub, which does not currently exist, would support more agile care delivery across the enterprise; maximize sharing of resources; facilitate implementation of standardization in sleep care nationally; and advance the country's vision for “One VA.”

## Data Availability

The raw data supporting the conclusions of this article will be made available by the authors, without undue reservation.
